# Aerobic Metabolic Adaptations in Endurance Eccentric Exercise and Training: From Whole Body to Mitochondria

**DOI:** 10.3389/fphys.2020.596351

**Published:** 2021-01-27

**Authors:** Julianne Touron, Frédéric Costes, Emmanuel Coudeyre, Hélène Perrault, Ruddy Richard

**Affiliations:** ^1^UCA–INRAE, Human Nutrition Unit, ASMS Team, University Clermont Auvergne, Clermont-Ferrand, France; ^2^Service de Médecine du Sport et des Explorations Fonctionnelles, CHU Gabriel Montpied, Clermont-Ferrand, France; ^3^Service de Médecine Physique et de Réadaptation, CHU Gabriel Montpied/CHU Louise Michel, Clermont-Ferrand, France; ^4^Respiratory Division, McGill University Health Center, Montreal, QC, Canada; ^5^Unité d’Exploration en Nutrition (UEN), CRNH Auvergne, Clermont-Ferrand, France

**Keywords:** oxygen consumption, free radicals, calcium, eccentric training, mitochondria

## Abstract

A characteristic feature of eccentric as compared with concentric exercise is the ability to generate greater mechanical loads for lower cardiopulmonary demands. Current evidence concurs to show that eccentric training translates into considerable gains in muscle mass and strength. Less is known, however, regarding its impact on oxygen transport and on factors to be considered for optimizing its prescription and monitoring. This article reviews the existing evidence for endurance eccentric exercise effects on the components of the oxygen transport system from systemic to mitochondria in both humans and animals. In the studies reviewed, specially designed cycle-ergometers or downhill treadmill running were used to generate eccentric contractions. Observations to date indicate that overall, the aerobic demand associated with the eccentric training load was too low to significantly increase peak maximal oxygen consumption. By extension, it can be inferred that the very high eccentric power output that would have been required to solicit a metabolic demand sufficient to enhance peak aerobic power could not be tolerated or sustained by participants. The impact of endurance eccentric training on peripheral flow distribution remains largely undocumented. Given the high damage susceptibility of eccentric exercise, the extent to which skeletal muscle oxygen utilization adaptations would be seen depends on the balance of adverse and positive signals on mitochondrial integrity. The article examines the protection provided by repeated bouts of acute eccentric exercise and reports on the impact of eccentric cycling and downhill running training programs on markers of mitochondrial function and of mitochondrial biogenesis using mostly from animal studies. The summary of findings does not reveal an impact of training on skeletal muscle mitochondrial respiration nor on selected mitochondrial messenger RNA transcripts. The implications of observations to date are discussed within future perspectives for advancing research on endurance eccentric exercise physiological impacts and using a combined eccentric and concentric exercise approach to optimize functional capacity.

## Introduction

Since its first description at the end of the 19th century (Fick, 1881), eccentric exercise has sparked much interest on account of distinctive features enabling high external mechanical power at reduced cost and its potential for positive rehabilitation and training outcomes (Hoppeler, 2016).

In regular daily movement, concentric and eccentric muscle contractions are combined, for example, when using the stairs or when braking a fall or lowering a heavy object. An activity that involves predominantly eccentric contractions can be described as an eccentric exercise, such as downhill running/walking or eccentric cycling, using a specifically designed ergometer (LaStayo et al., 2003). Endurance eccentric exercise is characterized by lower cardiorespiratory demands (Perrey et al., 2001; Minetti et al., 2002; Dufour et al., 2004; Chavanelle et al., 2014), which makes it particularly attractive for use in patients with chronic heart and lung diseases or with muscle impairments (Roig et al., 2008). Indeed, in individuals with a limited capacity to reach and sustain exercise intensities high enough for significant functional gains to be seen using traditional physical conditioning, the use of an eccentric modality with lower cardiorespiratory demand represents a valuable alternative. Moreover, a bonus outcome of eccentric training would be a non-negligible increase in muscle mass and strength, which is particularly interesting for individuals in whom skeletal muscle mass is compromised due to chronic disease, sarcopenia, and/or aging (Ellis et al., 2015; Cruz-Jentoft et al., 2019).

The impact of eccentric training on skeletal muscle mass and strength has been well-reviewed (Roig et al., 2009; Franchi et al., 2017; Julian et al., 2018a). However, the extent to which the framework for adaptations of the oxygen transport system remains the same as that for concentric exercise training stays unclear. To our knowledge, there has not been a comparative analysis of modality-specific elements that could potentially impact the training adaptation responses or serve as confounders in the interpretation of the data. This article aims to summarize and integrate findings relating to systemic adjustments to endurance eccentric exercise training with a particular emphasis on the central role of mitochondria for energy production and regulation of oxygen consumption (VO_2_). First, we review the specifics of endurance eccentric exercise and report on changes in maximal or peak oxygen consumption (VO_2_max or VO_2_peak) observed after eccentric exercise training. Second, we consider findings related to factors contributing to oxygen distribution and utilization in the context of acute and chronic eccentric exercise. Considering the dominant role of mitochondria for aerobic adaptations, we examine how eccentric exercise impacts global markers of skeletal muscle mitochondrial respiration and function modulators and regulators. Finally, we provide a perspective for current knowledge, gaps, and opportunities to devise and apply novel physical training and rehabilitation-based approaches.

## Distinctive Features of Endurance Eccentric Exercise

For the purpose of this review, we did not include studies that used repeated segmental resistance eccentric contractions but focused on studies in which dynamic eccentric exercise was carried out using adapted cycle ergometry or downhill walking/running with the view to enable whenever possible comparisons across modality with concentric work.

In humans, depending on the exercise modality, the metabolic cost of eccentric exercise has been reported as being half to one third that of a concentric exercise of equivalent mechanical power output (Perrey et al., 2001; Minetti et al., 2002). Conversely, the power output generated using an eccentric exercise modality can be more than twice that of concentric exercise for the same VO_2_. A similar observation has been reported in rats, using uphill or downhill treadmill running at given speeds and grades, showing the oxygen uptake during eccentric downhill running to be halved compared with concentric work (Chavanelle et al., 2014; Schlagowski et al., 2016). Although the lower oxygen cost of eccentric exercise has been shown repeatedly in both humans and animals, the kinetics of the cardiorespiratory responses through an incremental eccentric exercise protocol have not been as widely examined. In humans, only a few studies have compared concentric and eccentric physiological adaptations with volitional exhaustion probably because of the requirement to safely generate very high mechanical power output during the eccentric exercise protocol (Dufour et al., 2004; Lechauve et al., 2014; Lipski et al., 2018; Lemire et al., 2020). In previous studies of healthy, active young men, data were reported on eccentric vs. concentric heart rates (HR’s) and cardiac output across a wide range of mechanical power outputs as well as for incrementally increasing oxygen uptakes. Consistent with the lower oxygen cost per watt of eccentric mechanical power, cardiac output was significantly reduced on account of a lesser chronotropic effect (Dufour et al., 2004). Differences in respiratory adaptations were seen primarily with respect to ventilatory mode with an undifferentiated VO_2_–ventilation relationship being observed between concentric and eccentric exercise tests but with the latter characterized by a lower tidal volume and hyperpnea (Lechauve et al., 2014). In a more recent study, Lipski et al. (2018) have extended the range of comparison by monitored cardiopulmonary responses during incremental tests with exhaustion during both concentric and eccentric cycling. Similarly, results showed a down and rightward shift of the HR-power output response. However, the extension to task failure relationship enabled to establish that peak HR was not different between modalities but that peak power was 50% higher under concentric modality. Peak minute ventilation was also seen to be significantly lower during eccentric cycling, explained by a lower tidal volume, as peak breathing frequency was not different between modalities. A similar observation was made during uphill and downhill running where subjects reached the same maximal HR but failed to reach concentric VO_2_max value under eccentric exercise modality (Lemire et al., 2020). These results are of significance, as they provide a quantifiable comparison of cardiorespiratory demands that can serve to guide exercise rehabilitation recommendations. The extent to which physiological exercise adaptations translate to an improvement in peak aerobic capacity is however, highly intensity-dependent, which may be limiting on account of the greater skeletal muscle lesions induced by eccentric type contractions.

Compared with concentric muscle contractions, the lengthening effect of eccentric contraction results in a greater mechanical tensile force production leading to enhance exercise-induced muscle damage. Muscle damage can be classified according to the sequence of occurrence, with a first set, more immediate relating to the mechanical stress of exercise and another appearing subsequently as skeletal muscle cells react to the physical insult, such as cellular inflammation, intracellular calcium imbalance, or oxidative stress disruptions (Pereira Panza et al., 2015). In addition to reported symptoms of discomfort or pain, several histological and biological markers have been used to qualify and quantify the eccentric exercise impact. With respect to the impact of the mechanical constraints on the skeletal muscle structure, several studies have described morphological abnormalities such as disruptions of myofibrils and cytoskeleton and widespread Z-line streaming appearing gradually after eccentric exercise (Armstrong et al., 1983; Fridén and Lieber, 2001). Sarcomere disorganization could also be associated with extracellular matrix disruption, mitochondrial swelling, oxidative stress, and inflammation (Pereira Panza et al., 2015). The skeletal muscle disruptions impact performance with transient decrements in maximal force seen immediately after eccentric exercise and lasting several days to a week (Balnave and Thompson, 1993; Howell et al., 1993; Malm et al., 2004; Peñailillo et al., 2013; Hyldahl et al., 2017). In addition to histological alterations, symptoms of muscle soreness associated with increased intramuscular compartment pressure appear within 24 h of exercise and extend over several days. Exercise-induced damage confirmation showing maximal swelling occurring between 4 and 7 days post-exercise has been provided in both humans and animal muscles using ultrasound elastography, magnetic resonance imaging, and quantitative analysis approaches (McHugh, 2003; Lacourpaille et al., 2014; Fu et al., 2020). An elevation in blood creatine kinase activity 24–48 h post-exercise reflecting myofiber membrane disruption is also a consistent finding after eccentric exercise, the magnitude of increase being related to the exercise intensity (Balnave and Thompson, 1993; Malm et al., 2004; Klossner et al., 2007; Frimpong et al., 2019). Increases in messenger RNA (mRNA) expression of inflammatory markers have also been reported 3 and 24 h after a single 45-min bout of strenuous treadmill downhill running in recreationally trained men (Buford et al., 2009). The extent to which the degree of cytoskeletal mechanical stress and timing of the damage and repair cycle modulate the adaptive responses is not clear. This point is indeed illustrated by the report of transient alterations after one session of unaccustomed/acute eccentric exercise, which resolve spontaneously with rest, as well as evidence that after a few bouts, a repeated effect is seen such that post-exercise markers of damage are no longer seen (McHugh, 2003; Hyldahl et al., 2017).

In a recent review paper, Hyldahl et al. (2017) propose an integrated perspective on the time course of various sarcomere and membrane damage markers and remodeling after eccentric exercise. The schematics show functional and extracellular matrix disruption damage responses over an initial 3-day period, with structural remodeling responses extending to several weeks afterward. The diagram also illustrates the “repeated bout effect” defined by a lesser muscle fiber disruption and related symptoms upon a repeated bout of same intensity eccentric exercise (Nosaka and Clarkson, 1995; McHugh, 2003; Hyldahl et al., 2017). Although of progressively declining magnitude, the repeated bout protective effect appears to last over several weeks, the degree of protection being proportional to the intensity and to the degree of muscle damage resulting from the initial eccentric exercise bout (Hyldahl et al., 2017; Bontemps et al., 2020). It is of interest, however, that a repeated bout effect is seen even after a preconditioning exercise bout as short as 12 min (Pierrynowski et al., 1987) or exercise intensities as low as 40% VO_2_max [see table in Bontemps et al. (2020)]. The concept is also put to practice when designing the 1–2-week “familiarization or habituation” phase that is generally integrated within endurance eccentric exercise training studies. In such protocols, animal or human participants are submitted to successive bouts of eccentric exercise of progressively increasing intensity and/or duration, each bout generally being separated by a 24–48 h rest period, such as is the case in studies appearing in Table 1. The extent to which the “repeated bout effect” contributes to the adaptation occurring during the familiarization period of an eccentric training program is difficult to establish clearly. Results from a study in rats using a combined magnetic resonance imaging and skeletal muscle histopathological analysis to explore the kinetics of eccentric exercise-induced injury showed maximal disruption to be seen at day 2 post-exercise with gradual muscle repair continuing until at least day 7 (Fu et al., 2020). In contrast to most investigations of repeated bouts effects in which there is a long period separating an initial and a second exercise, familiarization is characterized by successive exercise bouts separated by short rest periods.

**TABLE 1:** Effects of endurance concentric or eccentric training on peak aerobic power.

References	Population Number per group and status Age per group (mean ± SD)	Concentric training protocol (bout duration (min) × frequency (x/week) × training period (weeks) @ intensity indicator) Eccentric training protocol (bout duration (min) × frequency (x/week) × training period (weeks) @ intensity indicator)				
			VO_2peak_ or VO_2max_			
	
			Pre-post training (%)			
			CON	Stats. Sign.	ECC	Stats. Sign.
**Human studies**						
Rooyackers et al., 2003	12 COPD patients CON: 59 ± 13 years ECC: 59 ± 10 years	CON: C intermittent; 20 min × 5x/week × 10 weeks @ SaO_2_ > 90% ECC: C mixed (intermittent CON: 20 min × 5x/week × 10 weeks @ SaO_2_ > 90% + ECC: 15 min × 5x/week × 10 weeks @ CON W_peak_)	0	NS	0	NS
Meyer et al., 2003	6–7 CAD patients CON: 56 ± 9 years ECC: 53 ± 8 years	CON: C; 30 min × 3x/week × 8 weeks @ ∼60% VO_2peak_ and/or 85% HR_peak_ (74–82 rpm) ECC: C; 30 min × 3x/week × 8 weeks @ ∼60% CON VO_2peak_ and/or 85% CON HR_peak_ (51–60 rpm)	+7.1	NS	+10.4	*
Gremeaux et al., 2010	7 CAD patients CON: 45 ± 5 years ECC: 53 ± 1 years	CON: C; 30 min × 3x/week × 5 weeks @ target HR of VT_1_ (50 rpm) ECC: C; 30 min × 3x/week × 5 weeks @ target HR of CON VT_1_ (20 rpm)	+4.6	**	+14.2	**
Casillas et al., 2016	21 CHF patients CON: 62 ± 9 years ECC: 64 ± 10 years	CON: C; 32 min × 3x/week × 7 weeks @ target HR of VT_1_ (60 rpm) ECC: C; 32 min × 3x/week × 7 weeks @ target HR of CON VT_1_ (15 rpm)	+11.6	**	+11.4	NS
Toyomura et al., 2018	8–10 healthy CON and ECC: 23 ± 2 years	CON: R (+ 10% grade); 20 min × 3x/week × 5 weeks @ target HR of CON lactate threshold ECC: R (−10% grade); 20 min × 3x/week × 5 weeks @ target HR of CON lactate threshold	+5	**	+2	NS
Lewis et al., 2018	8–9 healthy CON: 46 ± 8 years ECC: 40 ± 8 years	CON: C; 30 min × 2 x/week × 8 weeks @ 60% W_peak_ ECC: C; 30 min × 2x/week × 8 weeks @ 60% CON W_peak_	+9.5	*	+0.3	NS
Julian et al., 2018b	11–12 obese adolescents CON: 13 ± 1 years ECC: 14 ± 1 years	CON: C; 45 min × 3x/week × 12 weeks @ 70% VO_2peak_ (55–65 rpm) ECC: C; 45 min × 3 x/week × 12 weeks @ 70% CON VO_2peak_ (55–65 rpm)	+10.9	*	+16.6	*

Relative change in selected performance variable calculated from mean data provided or values taken from publication figures as: (post-value – pre-value/pre-value × 100). C, cycling; CAD, coronary artery disease; CHF, chronic heart failure; CON, concentric; COPD, chronic obstructive pulmonary disease; ECC, eccentric; HR, heart rate; NS, not significant; R, running; SaO_2_, arterial oxygen saturation; VO_2_, oxygen consumption; VT_1_, first ventilator threshold; W, workload. *p < 0.05; **p < 0.01.

To our knowledge, how the sequence and timing of neural, structural, and inflammatory responses are integrated to facilitate adaptations during such a period has not been fully described. Nonetheless, when monitoring post-exercise symptoms and subsequent exercising ability, participants report limited delayed muscle soreness and are capable of increasing force production (Pageaux et al., 2020). The fact that by the end of the familiarization period subjects are capable of achieving at least twice the volume of exercise performed in the initial week may be taken as evidence that if there is repeated skeletal muscle disruption, it is not sufficient to impair function.

## Effects of Eccentric Training on Peak Oxygen Consumption

Repeated large muscle mass dynamic exercise of sufficient intensity induces systemic adaptations that lead to an increase in VO_2_peak/max, which is a strong indicator of the aerobic, muscle, and functional cardiorespiratory capacities (Bassett and Howley, 2000). Its improvement after regular aerobic exercise of moderate–high intensity (≥60% of the initial VO_2_max/peak) is well documented in many different populations (Diaz-Canestro and Montero, 2019). Interestingly, such adaptations have not been consistently observed after eccentric training of large muscle masses, performed in healthy subjects and also in patients. Despite the growing interest in eccentric training, data on the systemic metabolic and cardiorespiratory adaptations to such training remain scarce. Table 1 summarizes an updated set of studies examining the impact of eccentric exercise training in most cases using cycling ergometry to contrast effects with those of traditional concentric cycling.

Three of the seven studies were conducted on healthy participants, whereas the four others included patients with chronic heart or lung disease and completed the exercise protocol extending between 5 and 10 weeks within the context of an exercise rehabilitation program. Findings from Table 1 reveal a significant increase in the selected functional performance marker (VO_2_peak/max) ranging between 5 and 10% in five of the studies after concentric training, whereas significant positive changes between 10 and 15% were seen in three of the studies after eccentric training (Meyer et al., 2003; Gremeaux et al., 2010; Julian et al., 2018b). When comparing the relative impact of eccentric with concentric, Meyer et al. (2003) found eccentric but not concentric training to result in an enhanced peak aerobic power in their sample of coronary artery disease patients. Similarly, although significant improvements were seen after rehabilitation using both modalities, a greater improvement was in the eccentric-trained group (Gremeaux et al., 2010). In contrast, in patients with chronic heart failure, Casillas et al. (2016) reported no significant improvement after eccentric training but a significant improvement in the concentric-trained group. When considering results from samples of healthy participants, statistically significant increases were seen in all three studies after concentric exercise training (Lewis et al., 2018; Toyomura et al., 2018) and only in one study after eccentric training (Julian et al., 2018b). Interestingly, an increase in peak power ranging from 10.3 to 23.0% is a common observation after all eccentric training programs summarized in Table 1. Other indications of physiological adaptive changes have also been reported. At the end of the 7-week pulmonary rehabilitation program, Rooyackers et al. (2003) observed an improvement in the alveolar–arterial O_2_ gradient at peak exercise, potentially reflecting some level of systemic adaptation. Similarly, a lower VO_2_ requirement (−0.55 L min^–1^) for given submaximal eccentric power output was seen in a small sample of healthy subjects (*n* = 6) after five training weeks (Knuttgen et al., 1982). A clear mechanistic explanation for this observation suggestive of enhanced bioenergetic efficiency has not been provided. Although a full review of biomechanical repercussions of eccentric exercise is beyond the scope of this article, it cannot be excluded that changes in the biomechanical properties of soft tissues and skeletal muscles consecutive to the repeated eccentric exercise bouts could impact the oxygen cost of the contraction (Peñailillo et al., 2015). Other predominant impacts of eccentric relative to concentric exercise training relate to impacts on muscle strength, mass, and body composition. Significant increases in strength-related parameters have been reported as a result of the low-intensity training protocols in several studies from Table 1 (Gremeaux et al., 2010; Casillas et al., 2016; Julian et al., 2018b; Lewis et al., 2018; Toyomura et al., 2018). Eccentric exercise training has also been seen to reduce body mass in the study of obese adolescents, which could contribute to the reported outcome on VO_2_max when expressed relative to total body mass (Julian et al., 2018b). Thus, an increase in peak VO_2_ is not a consistent finding after endurance eccentric training; experimental evidence support that it can induce skeletal muscle physiological adaptive responses and that the repeated increase in metabolic demand can have a systemic impact (Drexel et al., 2008; Nikolaidis et al., 2008; Paschalis et al., 2011).

A common feature of studies presented in Table 1 is the relatively low exercise training intensity, established on the basis of a pre-training concentric incremental exercise with a training intensity set above 70% peak HR or peak VO_2_ in only three studies (Meyer et al., 2003; Gremeaux et al., 2010; Julian et al., 2018b). In the case of studies on patients with chronic heart or pulmonary disease, eccentric exercise training intensity was set from prior incremental concentric tests for a target training HR corresponding to that below the first ventilatory threshold or approximately 60% of peak VO_2_ or 85% symptom-limited peak HR or above 90% SaO_2_. As discussed previously, this translates to a significantly lower cardiorespiratory demand compared with concentric training (Dufour et al., 2004; Lipski et al., 2018). It is, therefore, likely that given equivalent metabolic training overload, similar improvements would be seen across modalities. Indeed, when considering the three studies from Table 1 in which the highest target training intensities were used, a significant impact of eccentric training is seen (Meyer et al., 2003; Gremeaux et al., 2010; Julian et al., 2018b). The potential for endurance eccentric training to equally enhance peak oxygen uptake is also confirmed in animals. In a recent report, VO_2_max measured in rats submitted to a regular program of downhill running at −14° grade, 35 m⋅min^–1^, five times weekly for 6 weeks was found to be 24% higher than that of sedentary animals (Hahn et al., 2007). If converting this speed of running to gauge training intensity, 35 m⋅min^–1^ may be seen to be near-maximal if one refers to the maximal uphill and downhill treadmill running speeds sustained by rats at a grade of +15 and −15°, previously reported to be 70 cm⋅s^–1^ or 42 m⋅min^–1^ (Schlagowski et al., 2016). Thus, similar to that for concentric exercise training, inasmuch as the training protocol induces an adequate overload of the oxygen transport system, a training-induced effect to enhance its peak function could be expected. Clear comparative data to definitely establish that similarity of response is, however, lacking.

To date, only a few studies have examined the central and/or peripheral circulatory adaptations during endurance eccentric exercise (Meyer et al., 2003; Dufour et al., 2004), and none to our knowledge have explored the repercussions of eccentric *vs*. concentric exercise training of similar intensities to compare differences in training-induced adaptations. With respect to peripheral circulatory adaptations, only a few studies have assessed skeletal muscle capillarization and angiogenesis after eccentric exercise training and in two cases in the context of post-disuse rehabilitation in rats. Using a hind limb suspension and flat or downhill training remobilization model, Cornachione et al. (2011) found an increase in capillary density with both remobilization protocols. Similarly, after hind limb immobilization, 21 days of downhill treadmill running (−16°; 17 m⋅min^–1^) were seen to induce significant angiogenesis in excess of that resulting from passive limb stretch of similar duration (Benedini-Elias et al., 2014). Eccentric cycling has also been shown to be responsible for a 47% increase in capillary-to-fiber ratio after eight training weeks, whereas no change was reported after concentric (LaStayo et al., 2000).

Taken together, these observations may be taken to suggest that irrespective of the eccentric or concentric nature of the skeletal muscle contraction, chronic regular, sustained large muscle mass endurance exercise sufficient to produce the same magnitude of overload to the oxygen transport and utilization requirements would induce similar skeletal muscle adaptations. Consequently, similar to that seen after large muscle mass training using concentric exercise, adaptations in skeletal muscle mitochondrial density and activity would also be expected to follow from chronic eccentric training.

## Mitochondrial Responses and Adaptations to Eccentric Training

Over recent years, evidence has been accumulating that mitohormesis, the ability of the mitochondria to trigger significant adaptive response signaling pathways to restore and improve function, may operate to govern adaptations to aerobic training (Merry and Ristow, 2016). The extent to which the mitohormetic effects of aerobic exercise are expressed and translated across exercise training modalities remains incompletely documented, as is the interplay of effects on signaling factors and regulators modulating mitochondrial function (Walsh et al., 2001; Zoll et al., 2002; Mueller et al., 2011; MacMillan et al., 2017). Like any other cellular organelle, mitochondria can be studied in terms of protein content, RNA expression, or isolated enzymatic activities and integrated functionality. Mitochondrial function can be analyzed in isolated mitochondria (Frezza et al., 2007) and permeabilized fibers (Kuznetsov et al., 2008) from fresh muscle biopsy samples. The differences between these techniques are mainly based on 1) the amount of tissue and 2) the use of *in vitro* or *in situ* (intracellular architecture conservation) mitochondria. Both methods allow assessing several mitochondrial parameters, including respiratory function through O_2_ consumption, reactive oxygen species production (ROS), and calcium (Ca^2+^) uptake. For the sake of simplicity, in this review, the different parameters will be discussed separately, although they are interconnected (Brookes et al., 2004). In this section, we present data related to mitochondrial respiration in eccentrically trained skeletal muscles in both animal and human studies and examine how the chronic exercise intervention directly or indirectly impacts mitochondrial function through monitoring of modulating signals and regulators.

### Impact on Mitochondrial Respiration: Acute vs. Chronic Responses

The precise functioning of the mitochondrial respiratory chain has been extensively described (Brown et al., 2010), with reports on mitochondrial respiration properties generally considering a set of three common indicators: V_0_ or “state 4” for basal respiration, V_max_ or “state 3” for maximal adenosine diphosphate (ADP)-stimulated respiration, and the ratio of V_max_/V_0_ referring to the respiratory control ratio (RCR). It was only at the beginning of the 21st century that researchers became interested in the effects of eccentric exercise on mitochondrial energy metabolism showing contrasting effects of eccentric exercise on mitochondrial respiration. As can be seen from Tables 2, 3, a limited number of studies have examined the impact of endurance eccentric exercise or training in humans, which may be related, at least in part, to the need to perform skeletal muscle biopsies for muscle samples to be obtained.

**TABLE 2:** Effects of acute endurance eccentric exercise on mitochondrial respiration parameters.

References	Population Number per group	Eccentric training protocol (bout duration (min) @ intensity indicator) Experimental methods (muscles/technique)						
			Mitochondrial respiration markers relative					
			to substrates and measurement time post-exercise					
			(%Δ from baseline or from sedentary group)					
	
			V_0_	Sign.	V_max_	Sign.	RCR	Sign.
**Human study**								
Walsh et al., 2001	6 healthy volunteers	C: 30 min @ CON W_max_ (with rapid W ↑) *Vastus lateralis*/skinned fibers	P + M Imm.: −17.4 48 h:+4.3 96 h: −4.3	NS NS NS	P + M Imm.: −2.5 48 h:+6.6 96 h: −2.5	NS NS NS	P + M Imm.:+14.3 48 h: 0.0 96 h: −3.6	NS NS NS
**Animal studies**								
Molnar et al., 2006	8 Wistar rats	R (−7°): initial speed @ 8 m⋅min^–1^, ↑ every 5 min up to 30 m⋅min^–1^; then 4 min @ 30 m⋅min^–1^ and 1 min @ 40 m⋅min^–1^ until exhaustion Deep portion of quadriceps muscle/isolated mitochondria	P + M Imm.: −0.3 S Imm.: + 7.9	NS NS	P + M Imm.: + 6.9 S Imm.: + 3.2	NS NS	P + M Imm.: + 6.5 S Imm.: −2.7	NS NS
Rattray et al., 2011	8 Sprague-Dawley rats	R (−14°): 90 min (5 min run + 2 min rest) @ 20–30 m⋅min^–1^ *Vastus* muscle group (*vastus intermedius, vastus medialis*, and *vastus lateralis*)/isolated mitochondria	P + M Imm.: −17.9 2 h: −16.2 48 h: −28.0	NS NS NS	P + M Imm.: −16.2 2 h: −2.7 48 h: −45.9	* NS *	P + M Imm.: −1.5 2 h: + 11.8 48 h: −29.4	NS NS *
Rattray et al., 2013	8–9 Sprague-Dawley rats	R (−14°): 90 min (5 min run + 2 min rest) @ 20 m⋅min^–1^ Red muscles (*vastus intermedius, vastus medialis*, and *vastus lateralis*)/isolated mitochondria	P + M 48 h: −12.7	NS	P + M 48 h: −25.8	+	P + M 48 h: −12.2	+
Magalhães et al., 2013	10 CD-1 mice	R (−16°): total = 120 min (15 min gradual speed ↑; 75 min @ 25 m⋅min^–1^; 30 min @ 30 m⋅min^–1^) Hindlimb muscles (*soleus*, *gastrocnemius*, and quadriceps) + *spinotrapezius*/isolated mitochondria	P + M Imm.: + 7.0 48 h: −11.6 S Imm.: + 7.3 48 h: −8.0	NS NS NS NS	P + M Imm.: −27.1 48 h: −10.5 S Imm.:+6.0 48 h: −4.0	* NS NS NS	P + M Imm.: −33.1 48 h: −0.1 S Imm.: −9.1 48 h: −10.3	* NS NS NS

Relative change in mitochondrial respiration markers calculated from mean data provided or values taken from publication figures as: (post-value – pre-value/pre-value × 100). C, cycling; M, malate; NS, not significant; P, pyruvate; R, running; RCR, respiratory control ratio; S, succinate; V_0_, basal mitochondrial respiration (state 4); V_max_, maximal mitochondrial respiration (state 3, adenosine diphosphate stimulated state); W, workload. *p < 0.05, +*p* < 0.10.

**TABLE 3:** Effects of endurance chronic eccentric exercise on mitochondrial respiration parameters.

References	Population Number per group	Eccentric training protocol (bout duration (min) × frequency (x/week) × training period (weeks) @ intensity indicator) Experimental methods (muscles/technique)						
			Mitochondrial respiration markers relative					
			to substrates and measurement time after last training session					
			(%Δ from pre-training or from sedentary group)					
	
			V_0_	Sign.	V_max_	Sign.	RCR	Sign.
**Human study**								
MacMillan et al., 2017	4 COPD patients	C: 30 min × 3x/week × 10 weeks @ 4 × 60–80% CON W_peak_ (60 rpm) *Vastus lateralis*/skinned fibers	No data		G + M 96 h: −3.6 G + M + S 96 h:+10.9	NS NS	No data	
**Animal studies**								
Molnar et al., 2006	8 Wistar rats	R (−7°): week 1, 4 × 5 min (2 min rest); weeks 2–8, 6 × 2.5 min (1 min rest) × 5x/week × 8 weeks @ 20–35 m⋅min^–1^ Deep portion of quadriceps muscle/isolated mitochondria	P + M 48 h:+14.1 S 48 h:+3.1	* NS	P + M 48 h:+4.1 S 48 h: −2.3	NS NS	P + M 48 h: −8.7 S 48 h: −4.9	* NS
Rattray et al., 2013	8–9 Sprague-Dawley rats	R (−14°): 30 min (5 min run + 2 min rest) × 5x/week × 1 week @ 20 m⋅min^–1^ Red muscles (*vastus intermedius, vastus medialis*, and *vastus lateralis*)/isolated mitochondria	P + M 48 h: −4.7	NS	P + M 48 h: −8.2	NS	P + M 48 h: −1.4	NS
Isner-Horobeti et al., 2014	8–10 Wistar rats	R (−15°): 20–40 min × 5x/week × 1 or 4 weeks @ 20 m⋅min^–1^ *Vastus intermedius*/Skinned fibers	No data		G + M 12 h: −13.2 (1 week) 12 h: −12.4 (4 weeks) S 12 h: −11.8 (1 week) 12 h: −21.0 (4 weeks)	NS NS NS NS	No data	
Schlagowski et al., 2016	8 Wistar rats	R (−15°): 20–30 min × 5x/week × 4 weeks @ 60–80% of maximal speed *Vastus intermedius*/Skinned fibers	G + M + S 24 h: 0.0	NS	G + M + S 24 h: −2.2	NS	G + M + S 24 h: −4.0	NS

Relative change in mitochondrial respiration markers calculated from mean data provided or values taken from publication figures as: (post-value – pre-value/pre-value × 100). C, cycling; COPD, chronic obstructive pulmonary disease; G, glutamate; M, malate; NS, not significant; P, pyruvate; R, running; RCR, respiratory control ratio; S, succinate; V_0_, basal mitochondrial respiration (state 4); V_max_, maximal mitochondrial respiration (state 3, adenosine diphosphate stimulated state); W, workload. *p < 0.05.

Mitochondrial respiration is a dynamic phenomenon, but because of technical limitations, its quantification is not provided in “real-time,” which complicates the interpretation of intervention impact. In the study of exercise training effects, the difficulty remains to dissociate immediate post-acute from post-chronic exercise effects. Several studies conducted, most of them using an animal model, have examined the impact of an acute bout of eccentric exercise on mitochondrial respiration. Walsh et al. (2001) were the first to assess the immediate and delayed impact of a single cycling eccentric exercise bout on mitochondrial respiration parameters in healthy young men. Results showed no significant effect on V_0_; submaximal ADP stimulated respiration (V_submax_) and V_max_ immediately after exercise and at days 2 and 4 of recovery. There was also no significant difference in the ADP sensitivity index [(V_submax_ − V_0_)/(V_max_ − V_0_)] and RCR. As can be seen in Table 2, in the other studies completed in rodents, pyruvate–malate ADP-stimulated mitochondrial respiration was measured in hind limb muscle immediately and up to 48 h post-downhill running at a negative treadmill incline of −7 to −14° and speeds ranging between 20 and 40 m⋅min^–1^ (Molnar et al., 2006; Rattray et al., 2011, 2013; Magalhães et al., 2013). Compilation of results shows no significant changes being reported for V_0_. Data on V_max_ do not reveal a consistent pattern of impact. In the immediate post-exercise period, significant decreases of magnitude −16 and −27% are seen only in two of the experimental conditions across studies (Rattray et al., 2011; Magalhães et al., 2013). Of the four studies examining the post-48-h window, a significant decrease (−45.9%) was reported by only one group on two different occasions (Rattray et al., 2011, 2013). In both of these studies, the exercise protocol parameters were not at the extreme range or intensity and duration and therefore could not be responsible for inducing more severe muscle damage. It is therefore difficult to conclude from these results that the delayed muscle damage present 2 days post-exercise negatively affects the mitochondrial respiration capacity. This observation may serve as a basis to rule out the confounding effect that acute muscle damage impacts the results from exercise training studies.

In the study by MacMillan et al. (2017) patients with chronic obstructive pulmonary disease (COPD) were randomly assigned to either a concentric or an eccentric exercise training protocol to contrast the effects of a rehabilitation program consisting of three weekly 30-min sessions for 10 weeks at an intensity defined by the target HR corresponding to 60–80% of peak power output established from a concentric incremental test. For the eccentric training group, the target work rate was computed as four times the power output corresponding the 60–80% of measured concentric peak power. The first 2 weeks served as a familiarization period, with the eccentric intensity being set at 20–40% of concentric peak power sustained for 20 min in week 1 and for 30 min in week 2 (Table 3). The mechanical power performed during eccentric exercise bouts was threefold the concentric power output. Patients showed significant increases in leg muscle strength after eccentric but not after concentric training, whereas no differences in the various respiration states were observed (MacMillan et al., 2017). In contrast, significant changes in electron transport chain complexes were seen in the group trained through concentric cycling (MacMillan et al., 2017). In addition, Table 3 shows the small set of four studies having measured mitochondrial respiration in skeletal muscles of animals submitted to eccentric exercise training using downhill running on a motor-driven treadmill (Molnar et al., 2006; Rattray et al., 2013; Isner-Horobeti et al., 2014; Schlagowski et al., 2016). As can be seen, training duration including the familiarization period ranged between 5 and 8 weeks, for running speeds between 20 m⋅min^–1^ up to maximal (previously measured at approximately 40 m⋅min^–1^), at a 5-day per week frequency and with a negative treadmill grade between −7 and 15°. As can be seen, a significant change in V_0_ is not a consistent observation after either 24 or 48 h, whereas, for maximal mitochondrial respiration rates, a significant impact is not reported in any of the studies irrespective of the post-exercise timing of muscle sampling.

### Reactive Oxygen Species Production

It is well known that several factors, such as oxidative stress and calcium fluxes, act as regulators of mitochondrial respiration rates. ROS are mainly by-products of mitochondrial O_2_ respiration and electron transport, in particular at the level of complexes I and III (Inoue et al., 2003). Other major endogenous sources of ROS in skeletal muscle include nicotinamide adenine dinucleotide phosphate hydrogen oxidase and xanthine oxidase (He et al., 2016). In some non-physiological conditions, which may result in membrane potential (Δψ) or Ca^2+^ uptake changes, ROS homeostasis can be altered due to an imbalance between production and detoxification/antioxidant mechanisms (Jezek and Hlavatá, 2005). Excessive mitochondrial ROS production may lead to the opening of the mitochondrial permeability transition pores (mPTP) and to the alteration of the respiratory chain complex activity (Choksi et al., 2004). ROS have therefore been long thought to serve only as by-products of respiration that caused oxidative damage and contributed to aging, mitochondrial disorders, and cell death. However, recent evidence indicates that ROS produced in mitochondria are involved in various cell signaling processes (Brookes et al., 2004). ROS also play an important role in response to dynamic exercise, which is influenced by exercise type, intensity, duration, and training (He et al., 2016).

Although there is growing evidence that ROS may be involved in exercise-induced muscle damage (Close et al., 2004; Nikolaidis et al., 2008; González-Bartholin et al., 2019), there are only a few investigations have assessed mitochondrial ROS production rate in relation to eccentric exercise, mostly after an acute bout. In the mid-2000s, Molnar et al. (2006) were the first to examine whether eccentric exercise promoted mitochondrial ROS generation. They found that in response to acute eccentric exercise in rats (one exhaustive downhill running bout), muscle hydrogen peroxide (H_2_O_2_) production and back electron flow were reduced in state 4 measurements, and most of the ROS came from reverse electron transport through complex I. No modification was observed in state 3 measurements. Authors speculated that the low ROS production post-eccentric exercise could be explained either by a decrease in complex I activity as the main ROS generator within the electron transport chain or by a decrease in mitochondrial matrix superoxide dismutase content. However, none of these suggestions could be validated because both of these parameters were unaffected by acute eccentric exercise. Because ROS production is also greatly dependent on Δψ and electrochemical gradient and because uncoupling proteins (U) can affect these parameters, levels of UCP3 protein were also quantified. Unfortunately, the authors reported no change in this protein level. Another study showed that 30 min of continuous downhill running did not change UCP3 mRNA expression in the rat *plantaris* muscle, whereas its expression was decreased by 27.6% in *tibialis anterior* compared with the control group (no exercise) (Bryner et al., 2004). Molnar et al. (2006) also measured H_2_O_2_ generation in rats after 8 weeks of intermittent downhill training to determine whether the attenuation of muscle damage with eccentric training was related to a reduction in mitochondrial ROS generation or an adaptation of antioxidant enzymes. In the chronic eccentric exercise group, they observed lower H_2_O_2_ level in state 4 measurements with pyruvate–malate or succinate but no change in state 3 measurements. They hypothesized that this reduction was associated with increased mitochondrial protein content because they also found that citrate synthase activity was increased. As observed for the acute eccentric condition, they did not detect any change in UCP3 protein level in response to chronic eccentric training. However, this does not exclude the possibility that eccentric exercise might cause intrinsic changes in mitochondrial function, such as mild uncoupling, and prevent excess ROS production. When ROS production after concentric and eccentric training performed at similar mechanical power was compared, basal mitochondrial H_2_O_2_ production rates were not different between groups after 5 and 20 days of training, regardless of the studied muscle (Isner-Horobeti et al., 2014). However, in *vastus intermedius*, 20 days of eccentric training significantly increased mitochondrial H_2_O_2_ production and free radical leak. Based on these results, the authors suggested a functional adaptation to the mitochondrial electron transport chain rather than a change in mitochondrial density because H_2_O_2_ production increased in state 3 but was unchanged in state 4 measurements. Finally, a third study found no difference in muscle ROS production after 4 weeks of eccentric exercise alone and of combined eccentric and concentric exercise (Schlagowski et al., 2016), partially confirming the results of the previous study. In view of the observations mentioned earlier in multiple leg muscles and using different training protocols, it seems that eccentric exercise is not associated with increased ROS production. An improvement in antioxidant status cannot be totally excluded but will need to be further examined. The absence of major ROS production modifications may be taken to explain, at least in part, the absence of any significant reduction in mitochondrial respiration after eccentric training.

### Calcium-Related Outcomes

Disruptions in cellular structural integrity promote extracellular calcium fluxes that, in turn, may alter function. Considering the higher mechanical constraints exerted by eccentric exercise on skeletal muscle structures, the issue of calcium balance and the impact of eccentric exercise-induced muscle damage is of prime importance. The key role of Ca^2+^ as a regulator for many cellular processes, including contraction, protein synthesis, and degradation, is well known. Muscle cytosolic concentration is finely regulated by Ca^2+^ transport across the sarcolemma membrane, sarcoplasmic reticulum (SR), and mitochondria. Ca^2+^ homeostasis is also essential for proper mitochondrial functioning, as much evidences indicate that mitochondrial Ca^2+^ plays a role in mitochondrial oxidative phosphorylation regulation, ATP production, mitochondrial permeability transition, and cell death (Gunter et al., 2004; Giorgi et al., 2018). Whereas a low increase in mitochondrial Ca^2+^ concentrations enhances respiration and ATP production, higher matrix Ca^2+^ accumulation can induce mPTP opening, electrochemical proton gradient dissipation, respiratory chain dysfunction, and ultimately mitochondrial swelling, membrane disruption, and release of pro-apoptotic factors (Halestrap, 2009).

Duan et al. (1990) were the first to show the potential role of Ca^2+^ homeostasis alteration in the etiology of muscle fiber damage and mitochondrial dysfunction. In an animal study, muscle injury resulting from acute downhill walking was strongly and positively correlated with mitochondrial Ca^2+^ content (MCC). The reduction of mitochondrial Ca^2+^ accumulation with chelators attenuated muscle fiber injury, confirming the central role played by Ca^2+^ in promoting muscle damage (Duncan, 1978). As SR primarily regulates skeletal muscle intracellular Ca^2+^ concentration, SR Ca^2+^ uptake and SR Ca^2+^ ATPase (SERCA) activity were measured in human *vastus lateralis* after a cycling exercise (Enns et al., 1999). SR Ca^2+^ uptake was depressed during the recovery period and delayed after the exercise end. SERCA activity was increased during the first 2 days of recovery and returned to the pre-exercise level at day 6 post-exercise. SR functional characteristics (i.e., Ca^2+^ uptake and release, SERCA activity) were also significantly reduced in the red part of the *vastus lateralis* in rats after 90 min of downhill running (−16°) at 15 m⋅min^–1^ (Chen et al., 2007). However, the SR membrane integrity was not affected. The previous observations may be taken to reflect an SR defect possibly related to Ca^2+^ management. The rise in Ca^2+^ can, however, also be ascribed to stretch-activated Ca^2+^ channels (SAC’s) opening with eccentric muscle contraction (Allen et al., 2005). Incubation of muscles with SAC blockers after eccentric contraction abolished or significantly reduced myocyte Ca^2+^ accumulation, indicating the role of SAC’s in this process (Sonobe et al., 2008). It is, therefore, likely that SAC’s contribute, at least partly, to the rise in intracellular Ca^2+^ and muscle fiber damage seen after eccentric contractions. More than 20 years after the first publication on the link between Ca^2+^ homeostasis alteration and muscle fiber damage, Rattray et al. (2011) reported an increase in MCC after acute downhill running in rats. Concomitant with the elevated MCC, the authors also found that the mPTP sensitivity to Ca^2+^ was higher at the same time point after the stress event. Moreover, the elevation in skeletal muscle MCC at 48-h post-exercise was lower after an acute bout of eccentric exercise compared with the untrained condition (Rattray et al., 2013). The authors suggested a correlation between MCC and RCR because high Ca^2+^ level was associated with lower mitochondrial respiratory function. Finally, they confirmed the increased sensitivity of mPTP opening to Ca^2+^ after acute eccentric exercise while showing that training increased mPTP tolerance to Ca^2+^. A study in mice confirmed the increased susceptibility of mPTP opening to Ca^2+^ and the transient impairment of skeletal muscle mitochondrial function after downhill running (Magalhães et al., 2013).

These first insights into the links between Ca^2+^ homeostasis and eccentric muscle contractions indicate that unaccustomed muscle solicitation causes a transient and local Ca^2+^ elevation and increases mPTP sensitivity. The absence of calcium-related issue after several weeks of training seems to suggest muscle and/or mitochondrial adaptation (Rattray et al., 2013). Although it is still difficult to clearly identify the targets of this adaptive response, the transient alteration in mitochondrial O_2_ consumption may be explained by the transient elevation of and sensitivity to Ca^2+^.

### Messenger RNA Expression of Mitochondrial Proteins

Over the last decades, several studies have examined the expression of mRNA after eccentric exercise to understand better molecular mechanisms underlying structural and functional changes induced by this training modality (Zoll et al., 2006; Klossner et al., 2007; Hody et al., 2011; Mueller et al., 2011; Valladares-Ide et al., 2019). Evidence from the literature indicates that “strength” exercises and “endurance” exercises do not activate the same gene networks and signaling pathways (Coffey and Hawley, 2007; Egan and Zierath, 2013). As reviewed, the PI3K-AKT-mTOR pathway is the main integrator of mechanical stress for hypertrophy and muscle mass gain, whereas endurance-type training induces the AMPK-p38 MAPK-PGC1α pathway, leading to mitochondrial biogenesis. As eccentric exercise and training are carried out in endurance mode, they would concomitantly involve significant mechanical stress and metabolic stimulation. However, to date, there are only scarce data on gene expression of mitochondrial function modulators and regulators after such stimulus.

The first study investigating the molecular response to eccentric endurance training was conducted in patients with coronary artery disease (Zoll et al., 2006). Analysis of steady-state adaptations of *vastus lateralis* after an 8-week training program of 30 min three times per week up to 60% of concentric VO_2_peak showed that the mRNA levels of mitochondrial transcription factor A and of cytochrome c oxidase subunit 4 were reduced after low-intensity eccentric training compared with concentric training. Conversely, PGC1α, a transcriptional coactivator central to mitochondrial biogenesis, was unchanged. Consistent with these findings, a study on the effects of a single bout of unaccustomed eccentric exercise (15 min at 50% of individual concentric maximal power output) later reported that the expression of 80 genes was significantly changed, mainly downregulated immediately after the bout and/or during the recovery time (Klossner et al., 2007). The authors concluded that the eccentric stimulus was insufficient to induce carbohydrate or lipid metabolism upregulation or to positively affect energy generation and mitochondrial biogenesis. Similarly, in older adults, after 12 weeks of eccentric training, only 29 transcripts were significantly changed (Mueller et al., 2011). Further examination showed genes encoding for metabolic enzymes and mitochondrial proteins were downregulated, whereas genes encoding factors involved in repair and remodeling were upregulated. Finally, a study on patients with COPD did not find any change in PGC1α expression and also in the electron transport chain complex content after a 10-week eccentric program (MacMillan et al., 2017).

Thus, the few studies currently available suggest that mitochondrial biogenesis is not stimulated in response to either acute or chronic eccentric exercise, at least when performed at intensities as used in studies summarized in Tables 1–3, which is consistent with the absence of changes in mitochondrial respiration parameters.

## Discussion

### Main Findings

Metabolic adaptations to eccentric training remain incompletely understood, but the currently available data indicate that eccentric exercise training as currently done does not induce adaptations in peak aerobic power and mitochondrial function. Spontaneous acute eccentric exercise may cause transient alterations in mitochondrial O_2_ consumption, Ca^2+^ matrix content, and sensitivity to Ca^2+^, with evidence for repeated exercise to provide some protective effect from these outcomes.

The observed lack of adaptive responses may be explained by the relatively low exercise training metabolic stimulus. This results in a dominance of the mechanical vs. metabolic overload induced by the eccentric exercise protocols and, in turn, in an uncoupling of the mechanical load and systemic metabolic demands on exercised muscles leading to a suboptimal metabolic stimulus (Meyer et al., 2003; MacMillan et al., 2017). This is supported by the absence of upregulation of mitochondrial and metabolism-related genes, although a significant increase in muscle mass and strength are reported after the eccentric exercise training, presumably the outcome of the activation of these specific signaling pathways attesting to the capacity of response.

### Perspectives for Functional Capacity Recovery and Rehabilitation

Because of the high mechanical power output to low cardiorespiratory demand advantages conferred by the eccentric-type contractions, its use for restoring muscle function after an acute injury or enhancing mobility and exercise capacity in individuals with chronic heart or lung disorders is increasingly recognized. This modality is safe and effective for patients with chronic diseases (LaStayo et al., 2007; Rocha Vieira et al., 2011; Besson et al., 2013; Pageaux et al., 2020). However, in addition to the practical difficulties of defining and monitoring training parameters to minimize muscle damage and soreness, the use of eccentric exercise for rehabilitation remains a challenge for several reasons. First, mechanical loads required to overload the oxygen transport system in a way sufficient to induce desired improvements may be too high to be sustained by patients. Second, because of this disproportional coupling of mechanical and metabolic loading, it is difficult to design an eccentric exercise training protocol that can adequately address the overall functional improvement. Except in cases where muscle strengthening is the predominant goal and in which cases, targeted segmental eccentric exercise might offer advantages over traditional resistance training, the use of endurance eccentric exercise needs to be considered in combination with other approaches.

Various technological advances are being made to allow for more selectivity in nature and in the sequence of loading across movement cycles (Flück et al., 2017; Franchi and Maffiuletti, 2019; Doyle et al., 2020). As more technological advances are being made, so are opportunities for a more “individualized” approach to exercise rehabilitation. This requires an understanding of not only the targeted physiological and/or functional adaptive responses expected from the “designer” program but also an appreciation of the selection and the combination of “building blocks” that will best serve the identified purposes. In theory, the best combination should be one to optimize the overall physical training outcomes, enhancing muscle strength, and endurance and aerobic capacity. In practice, this will require more advanced knowledge of the impact of varying eccentric exercise modalities on skeletal muscle structures and ultra-structures to understand the fine balance between disruptive and adaptive responses to exercise. A wide range of possible combinations should be integrated to include blocks of segmental isokinetic and/or isoweight eccentric strength training, large muscle mass eccentric/concentric exercise aerobic training, allowing for blocks of different duration, sequencing, and stacking. Similarly, within each block, consideration can be given to use continuous and discontinuous approaches, with loading strategies to include periodization and various combinations of constant load or intermittent exercise in alternating sequences (Flück et al., 2017; Harris-Love et al., 2017; Franchi and Maffiuletti, 2019; Plaquevent-Hostache et al., 2019).

As expressed in the previous sections, exercise-induced muscle damage may be seen as a necessary evil to trigger skeletal muscle adaptations. In the same manner, as “mitohormesis” is used to define the delicate balance of negative and positive influences of ROS on mitochondria, a concept of “eccentric hormesis” could be introduced to capture the duality of deleterious and beneficial effects particularly associated with this exercise modality. Although there is growing evidence that muscle growth can be triggered by alternative pathways (Fukada et al., 2020), muscle destabilization as induced by eccentric contraction allows adaptations, whether or not it is accompanied by symptoms of muscle damage. In applying this concept, building blocks can be selected with the view to modulate the exercise prescription such that the exercise-induced muscle damage is of nature and magnitude just enough to trigger a beneficial adaptation but not to the extent that it has a deleterious effect that prevents adaptive activation or impairs performance. From the current review, it would appear that in aligning building blocks, a predominance of moderate–high intensity dynamic concentric components would be needed to impact skeletal muscle mitochondrial function. A combination of traditional aerobic type training and segmental eccentric strength could enable optimization of overall rehabilitation impact. The higher the proportion of eccentric contractions, the higher the signals for muscle mass and strength building but presumably at the expense of mitochondrial adaptations and peak aerobic function effects. On the other hand, if the proportion of eccentric exercise-induced damage is too low to activate the signaling pathways, the result could be a loss of impact on skeletal muscle and body composition without impact on oxygen transport or utilization.

Although experimental evidence is still needed to validate many aspects of the eccentric-hormesis approach, an example focusing on exercise prescription from the rehabilitation setting is presented here for illustration purposes. Among patients with chronic heart or lung disease whose progression is such that there is very pronounced and significant muscle atrophy, the prescribed balance would favor the eccentric exercise component to stimulate muscle mass and strength gains, even if at the expense of mitochondrial function adaptations. In such patients, endurance eccentric exercise carried out at a low% VO_2_peak would remain well within limits imposed by factors of chronotropic insufficiency, low cardiac-output, or ventilatory restrictions. After some 4 or 5 weeks, as previously seen in COPD or coronary artery disease (Meyer et al., 2003; MacMillan et al., 2017), the eccentric load maintained by patients would start approaching levels of metabolic demands susceptible to trigger aerobic-related effects. Resetting exercise prescription recommendations would then focus on gradually reaching exercise levels equivalent to or higher than 60% VO_2_peak with the view to maintaining strength gains while minimizing exercise-induced muscle soreness. These goals could be implemented through different combinations of alternating, sequential, or stacked blocks of continuous or intermittent concentric training, eccentric segmental strength maintenance exercises with gradual re-shifting toward higher eccentric loading as dictated by the patient’s limits and progression.

The importance of regular exercise for the maintenance and rehabilitation of individuals with physical limitations is no longer a debate. Several research teams are contributing conceptual, theoretical, and technological bases for approaches to enhance the impact and effectiveness of exercise prescription. New tools and technologies continue to be developed and need to be tested, promoted, and better integrated into non-research or institutional settings. Approaches to gauge symptoms of soreness and overall patient well-being also need to be refined to understand better how the information is best integrated to center around the willingness, the ability, and the enthusiasm of the patient to sustain the exercise prescription.

## Author Contributions

JT, FC, EC, HP, and RR edited the English version of the manuscript. All authors contributed to the article and approved the submitted version.

## Conflict of Interest

The authors declare that the research was conducted in the absence of any commercial or financial relationships that could be construed as a potential conflict of interest.
